# Research progress and narrative review of totally implantable venous access ports

**DOI:** 10.3389/fonc.2026.1800754

**Published:** 2026-04-24

**Authors:** Andong Yu, Ruochong Pang, Mingqing Zhang, Liang Yin, Xuming Bai, Yong Jin, Xingwei Sun

**Affiliations:** Department of Interventional Radiology, The Second Affiliated Hospital of Soochow University, Suzhou, Jiangsu, China

**Keywords:** central venous access, clinical application, complication management, evidence-based progress, totally implantable venous access port

## Abstract

Totally Implantable Venous Access Ports (TIVAP) are specialized devices designed for long-term venous therapy, widely used in oncology and other groups requiring prolonged intravenous access. Since their introduction into routine clinical practice in China, they have significantly enhanced the safety and comfort of vascular access management. However, substantial challenges remain in TIVAP utilization as China lacks a unified regulatory framework and standardized full-lifecycle management protocols. Significant disparities also exist among medical institutions in terms of surgical techniques, complication management, and surveillance, leading to inconsistent outcomes and patient satisfaction. This narrative review presents a review of TIVAP clinical applications and maintenance, incorporating the latest evidence-based strategies for implantation and complication mitigation. The objective is to provide a comprehensive reference for optimizing clinical decision-making, minimizing adverse events, and improving patient quality of life.

## 1 Introduction

With the rapid advancement of oncology therapy and critical care medicine, establishing reliable, durable long-term venous access has grown increasingly indispensable in modern clinical practice (1). As a fully implantable venous access system, TIVAP has steadily become the preferred modality for prolonged venous therapy since its initial inception in the 1970s, offering advantages such as complete subcutaneous implantation, minimal external exposure, ease of use, and reduced infectious risk (2, 3). Despite the extensive clinical application of TIVAP, several critical challenges persist in the management of oncology patients. First, the incidence of catheter-related complications remains high; second, there is a lack of unified standardization in implantation procedures, particularly regarding the brachiocephalic vein approach; and third, significant inter-institutional variability exists in the protocols for managing postoperative complications (4). This review comprehensively synthesizes the latest clinical evidence on TIVAP, covering the selection of optimal implantation vasculature, precision catheter tip positioning technologies, and evidence-based strategies for complication management, with the aim of providing a solid scientific foundation for clinical practice.

### 1.1 Literature search strategy for TIVAP-related research

To achieve a rigorous and evidence-based synthesis for the objectives outlined above, a prioritized literature search was conducted across major international databases, including PubMed, Embase, Web of Science, and the Cochrane Library. We focused on high-quality evidence published between January 2015 and December 2025, utilizing a combination of Medical Subject Headings (MeSH) and free-text keywords such as “Totally Implantable Venous Access Port, “ “TIVAP, “ “brachiocephalic vein, “ “innominate vein, “ and “complication management.”

Given our institution’s pioneering role in the ultrasound-guided brachiocephalic vein (BCV) approach since 2017—an area extensively documented in our international peer-reviewed publications—this specific technique was a focal point of our evaluation. The selection process followed a structured hierarchy, prioritizing multi-center randomized controlled trials (RCTs), high-impact meta-analyses, and the latest international standards, such as the INS 2024 Infusion Therapy Standards of Practice. Ultimately, 62 high-quality publications—representing both global evidence and our internationally-recognized institutional experience—were integrated to provide the scientific foundation for this review.

## 2 Structure of TIVAP

The Totally Implantable Venous Access Port (TIVAP) is a fully implantable venous access system designed for long-term clinical use. It fundamentally consists of two primary components: (1) an injection port (reservoir) implanted subcutaneously; (2) a radiopaque venous catheter, with its distal tip typically positioned in the superior vena cava (SVC)While a dedicated non-coring needle (Huber needle) is essential for accessing the system, it is considered an external clinical tool rather than an intrinsic part of the implanted device.

The injection port features a high-density, medical-grade silicone septum with self-sealing properties, capable of withstanding repeated punctures without compromising integrity. The port base is typically constructed from medical-grade titanium alloy or biocompatible polymers to ensure compatibility with Magnetic Resonance Imaging (MRI) and Computed Tomography (CT) scans. Catheter material selection is critical for ensuring biocompatibility and mechanical durability. Currently, two primary catheter types dominate clinical practice: silicone catheters, which offer excellent flexibility but are relatively prone to kinking and gradual degradation over time, and polyurethane catheters, which boast enhanced pressure resistance and durability but come with higher cost implications (5).

## 3 Indications and contraindications of TIVAP

### 3.1 Indications

TIVAP is indicated for several distinct clinical scenarios: (1) Patients requiring long-term intravenous infusion of corrosive, irritant, or hyperosmolar therapeutic agents—such as oncology patients undergoing neoadjuvant, adjuvant, or palliative chemotherapy regimens—where TIVAP helps effectively mitigate peripheral venous injury and ensures the uninterrupted delivery of treatment; (2) Individuals in need of prolonged parenteral nutrition support, for whom TIVAP provides a stable and reliable vascular access to meet their essential metabolic demands; (3) Patients with compromised peripheral vascular integrity who require recurrent blood sampling or frequent drug administration, as TIVAP alleviates the discomfort and potential risks associated with difficult peripheral venipuncture; (4) Individuals requiring chronic transfusion of blood products, where TIVAP facilitates safe, efficient, and minimally invasive transfusion procedures (6, 7).

Notably, while no universal consensus has been reached (8).

Notably, no universal consensus has been reached regarding the precise temporal definition of “long-term” therapy, contemporary standards provide clearer frameworks for clinical decision-making. The INS 2024 Infusion Therapy Standards of Practice and ESPEN guidelines generally advocate for TIVAP placement when treatment is expected to exceed 3 months, though some literature suggests a threshold as low as 4 weeks for certain vesicants. Beyond these timeframes, clinical evidence consistently confirms that TIVAP delivers superior cost-effectiveness compared to conventional peripheral or non-tunneled central venous catheters, particularly when the treatment course exceeds 6 months (8–10). For indwelling durations beyond 12 months, TIVAP further outperforms alternative access devices in both cumulative cost-efficiency and key clinical outcome metrics, such as reduced infection rates and improved patient quality of life. Thus, the recommendation for TIVAP increasingly integrates both a 3-to-6-month temporal baseline and the specific pharmacological properties of the therapy (11, 12).

### 3.2 Contraindications

The contraindications for TIVAP implantation are categorized into absolute and relative constraints to ensure patient safety and procedural success, in accordance with the latest international and national standards (9).

#### 3.2.1 Absolute contraindications

(1)Systemic Infection: Presence of sepsis or bacteremia, which significantly elevates the risk of Catheter-Related Bloodstream Infection (CRBSI) (9, 13); (2)Localized Infection: Active infection at the intended pocket site or along the projected venous track; (3)Uncorrectable Coagulopathy: Severe bleeding diathesis or coagulation disorders that cannot be managed clinically prior to the procedure (10); (4)Material Hypersensitivity: Confirmed allergy to device components, such as medical-grade silicone, titanium alloy, or specialized polymers (9).

#### 3.2.2 Relative contraindications

(1)Correctable Coagulopathy: Moderate hematological abnormalities, typically defined as a platelet count < 50 ×10^^9^/L or an INR > 1.5. Implantation may proceed once these parameters are optimized via transfusion or medical intervention (9);(2)Anatomical Obstruction: History of Superior Vena Cava (SVC) Syndrome, localized venous stenosis, or previous Deep Vein Thrombosis (DVT) in the target vessel (10); (3)Severe Cardiopulmonary Dysfunction: Patients unable to tolerate a supine position or the physiological stress of the surgical procedure; (4)Extreme Cachexia: Insufficient subcutaneous tissue (typically < 0.5cm of fat) to adequately cover the port reservoir, increasing the risk of skin erosion or port dehiscence (9).

## 4 Selection of TIVAP access routes

### 4.1 Chest wall TIVAP access

#### 4.1.1 Internal jugular vein access

The internal jugular vein remains a gold-standard access route for TIVAP implantation, owing to its substantial diameter (mean 10–15 mm), fixed anatomical location, and distinct surface landmarks. The widespread use of ultrasound-guided puncture has significantly optimized procedural outcomes, enabling real-time visualization of vascular anatomy to minimize iatrogenic injury. However, this route has inherent limitations: the sharp 180-degree turn required to reach the subclavian region predisposes long-term indwelling catheters to mechanical stress-induced kinking, thrombosis, or fibrin sheath development. Furthermore, the cervical puncture site and subcutaneous tunnel may cause local discomfort or traction pain during active cervical movement. Clinical data consistently show that while the long-term complication rate of the internal jugular vein approach (9.87%) is lower than that of the subclavian vein (17.68%) (14), vigilant monitoring for catheter-related infections (2.53%–8.2%) and potential vascular trauma is essential (15, 16).

#### 4.1.2 Subclavian vein access

Subclavian vein puncture is characterized by its directanatomical trajectory and relative operational simplicity, with better puncture site concealment than the internal jugular vein. However, its anatomical proximity to the pleural apex and subclavian artery elevates therisk of pneumothorax, hemothorax or neurovascular injury. Additionally, catheter passage between the clavicle and first rib makes it prone to mechanical compression, triggering “pinch-off syndrome” (POS), with an incidence of 2.60%–3.45%, which clinically presents as catheter occlusion, structural rupture, or impaired infusion efficacy (17). Furthermore, the incidence of mechanical complications in the subclavian vein approach (9.70%) is significantly higher than in the internal jugular vein (3.75%), due to increased susceptibility to catheter displacement during routine movements (18). Despite lower catheter occlusion rates in pediatric populations, the overall complication rate (7.28%) still limits its widespread application in adult patients (19).

#### 4.1.3 Axillary vein access

Since Westcott first described axillary vein puncture in 1972, this access route has emerged as a preferred alternative due to its favorable safety profile and enhanced patient comfort. The third segment of the axillary vein, located deep to the pectoralis major muscle, forms a natural anatomical angle with the chest wall, effectively reducing the risk of pneumothorax (incidence < 1%). Its linear catheter trajectory—obviating the necessity for a lengthy subcutaneous tunnel—minimizes cervical traction and lowers infectious complications (20, 21). However, the axillary vein exhibits significant anatomical variability (such as individual differences in depth and branching patterns), requiring real-time ultrasound guidance and high operator proficiency to ensure successful puncture.

#### 4.1.4 Brachiocephalic vein access

Following the growing international interest in supraclavicular access, Sun et al. pioneered the clinical application of right brachiocephalic vein(BCV) TIVAP implantation in China in 2017, introducing an innovative and promising approach for long-term venous access in oncology patients (22). In 2019, the team further systematically validated the safety and efficacy of ultrasound-guided right BCV TIVAP implantation in large cancer patient cohorts (23). These findings align with global trends, such as those established by Simonov et al., who have advocated for the BCV as a primary alternative to the subclavian vein to avoid mechanical complications (7).

Contemporary clinical evidence indicates that ultrasound-guided BCV(also known as the innominate vein, INV) access achieves a puncture success rate of 92.52%–98.53% with a low complication rate of 4.67%–6.24%. This approach demonstrates significant efficacy in reducing catheter malposition, Pinch-off Syndrome (POS), and other adverse clinical events. Moreover, integrating real-time intraoperative ultrasound guidance has cut the perioperative complication rate to 1.15%–1.18%, which is significantly lower than the rate associated with traditional anatomical landmark-guided puncture. Clinical studies have confirmed a 71% reduction in complication rates compared to blind puncture techniques (23–25). A comparative study by Liu et al. showed that the BCV group had the shortest intravascular catheter length, higher 1-month postoperative comfort scores, and no reported cases of POS—unlike the internal jugular vein and subclavian vein groups (26).

The clinical utility of BCV access is further supported in specialized populations. Ding et al. extended these findings to pediatric populations, confirming the safety and efficacy of BCV access in children requiring long-term chemotherapy or recurrent blood transfusion (27). This is consistent with the seminal work of Thompson, whose prospective studies in Europe established the BCV as a safe and high-success-rate vessel for central venous access in neonates and infants (28).

Growing clinical evidence from both domestic and international sources strongly supports the BCV as a promising preferred access route for TIVAP implantation (23–25). However, left BCV puncture still carries a potential risk of thoracic duct injury, with relatively limited clinical data available to fully assess this risk. Further prospective randomized controlled trials are therefore needed to standardize protocols and validate these preliminary observations.

### 4.2 Arm TIVAP access

The clinical use of arm TIVAP was initially reported in international academic literature by Winters et al. in 1990 (29), Subsequently, the Infusion Nurses Society (INS) formally recognized it in the 2016 and 2021 “Infusion Therapy Standards of Practice” as a viable and effective alternative to chest wall TIVAP (30). Arm venous access primarily utilizes the basilic, brachial, or cephalic vein as preferred puncture paths, with insertion guided by standardized ultrasound-guided Seldinger technique. The catheter tip is precisely positioned at the cavoatrial junction (CAJ), and the port is implanted subcutaneously on the lateral or medial aspect of the arm, tailoring the catheter length (typically 30–45 cm) to the patient’s morphometric measurements (31, 32).

Comparative clinical trials have consistently demonstrated that arm venous access carries a significantly lower risk of mechanical complication than internal jugular vein and subclavian vein access, with a pneumothorax incidence of nearly zero and no reported cases of POS. While early concerns focused on Upper Extremity Venous Thrombosis (UEVT), recent multi-center studies by Bertoglio et al. indicate that thrombosis rates are non-inferior to chest wall access, with rates as low as 1.8% achievable through refined micro-incision techniques (32, 33). Compared to chest wall TIVAP, the arm port features a minimal pocket incision (1.5–2 cm). Postoperative pain is notably milder, further enhancing patient recovery. Additionally, the concealed port location and lenhanced privacy during infusion therapy contribute to and higher patient acceptance rates and improved body image scores (33, 34).

Despite its advantages, the arm approach presents specific technical challenges. The initial puncture failure rate for inexperienced operators is approximately 7.5%, and the catheter displacement rate ranges from 0.3% to 1.7%, requiring accurate tip localization via intraoperative fluoroscopy or Digital Subtraction Angiography (DSA) (24, 32). While the overall infection rate (4.9%) is comparable to chest wall access, specific complications such as the incidence of port rotation (0.4%–0.5%) and transient limb discomfort (≈1.1%) may occur (32, 35). As a critical alternative, arm TIVAP is widely indicated in patients with chest wall contraindications, including prior radical mastectomy, chest wall radiotherapy, or severe tracheostomy, providing a flexible and valuable clinical solution.

### 4.3 Selection of catheter implantation technology

With the evolving paradigm of minimally invasive surgery, modern vascular access technology has undergone a transformative shift from traditional open surgical approaches to advanced image-guided interventional techniques. The Seldinger puncture technique—a cornerstone of interventional radiology—offers distinct advantages by establishing a guidewire-assisted working channel, substantially reducing the risk of vascular trauma and related complications (36). In recent years, the widespread clinical adoption of real-time ultrasound (US) guidance has significantly improved the success rate and safety profile of percutaneous puncture and catheterization, firmly establishing it as the first-line clinical modality for TIVAP implantation. According to the 2024 Infusion Therapy Standards of Practice, US guidance is now firmly established as the “gold standard” and first-line clinical modality for TIVAP implantation, effectively eliminating the risks associated with landmark-guided “blind” puncture (9, 36).

### 4.4 Catheter tip positioning technology

#### 4.4.1 Anatomical positioning technology

The current guidelines of the Infusion Nurses Society (INS) explicitly define the optimal catheter tip position as the lower third of the superior vena cava (SVC) near the cavoatrial junction (CAJ). In this specific anatomical region, blood flow velocity exceeds 2000 mL/min, facilitating rapid drug dilution and minimizing venous irritation (37, 38). Anatomical studies have confirmed significant interindividual variability in the correlation between surface landmarks and the CAJ (39). Two standardized anatomical positioning methods are commonly utilized: (1) Formulaic prediction: Expected catheter insertion length = (patient height in cm)/10 − 3; (2) Anatomical landmark measurement: Reference distance from the puncture site to the right sternoclavicular joint plus a 7 cm compensation length (24, 40). Due tomorphological diversity and patient positioning shifts, anatomical positioning alone has suboptimal accuracy, often requiring intraoperative verification.

#### 4.4.2 Imaging-guided positioning technology

Real-time ultrasound navigation: Transthoracic echocardiography (TTE)-guided positioning enables dynamic and continuous visualization of the catheter tip, with multicenter clinical studies showing significantly higher accuracy than traditional surface measurement techniques. Micro-intracardiac echocardiography (ICE) catheters provide real-time anatomical visualization of the CAJ and surrounding structures, reducing the need for repeated adjustments (41).

X-ray positioning navigation: Postoperative chest X-ray remains the standard clinical modality for final catheter tip confirmation, using the tracheal carina as the primary reference to infer the CAJ position. Intraoperative fluoroscopy offers real-time positional monitoring, acting as a practical alternative in interventional suites (42). Digital Subtraction Angiography (DSA): Contrast-enhanced DSA provides high-precision visualization of the catheter tip and its spatial relationship with the surrounding vasculature, offering accuracy in complex clinical cases (43).Electromagnetic navigation system: Integrating electromagnetic induction and three-dimensional reconstruction technology, this innovative modality achievesprecise tip tracking, making it particularly suitable for patients where ionizing radiation should be minimized (44).

#### 4.4.3 Electrophysiological positioning technology

Intracavitary electrocardiography (IC-ECG) assesses catheter tip position based on characteristic changes in P-wave morphology. As established by the GAVeCeLT consensus and studies by Simonov et al., the emergence of the “maximal P-wave” serves as a highly accurate marker for the CAJ (7). As aradiation-free alternative to fluoroscopy, IC-ECG is widely used internationally to provide real-time, cost-effective, and precise tip localization during the procedure (45). This technique effectively reduces the reliance on postoperative X-rays and minimizes the cumulative radiation dose for oncology patients.

## 5 Complications of TIVAP and evidence-based management

The overall incidence of TIVAP-related complications ranges from 1.8% to 14.4% in clinical practice While standardized protocols are essential for prevention, timely diagnosis and structured management are critical for mitigating adverse outcomes (46). Complications are typically categorized by their temporal onset: intraoperative complications, early postoperative complications (≤30 days), and late postoperative complications (>30 days). A comprehensive summary of these complications, including their incidence and evidence-based management strategies, is presented in **Table 1**.

**Table 1:** Summary of clinical complications, incidence, and management strategies for TIVAP.

Phase	Complication	Incidence (%)	Primary Etiology/Risk factors	Management & prevention
Intraoperative	Pneumothorax	0.2% – 1%	Accidental pleural or lung injury; operator inexperience.	Observation/oxygen for stable cases; drainage for severe cases.
	Vascular Injury	~1%	Accidental arterial puncture; multiple blind attempts.	Manual compression; use of real-time ultrasound guidance.
	Air Embolism	Rare	Air entry via open needle/sheath; patient tachypnea.	Left lateral decubitus & Trendelenburg; high-flow oxygen.
	Arrhythmia	N/A	Stimulation of the endocardium by guidewire/catheter.	Continuous ECG monitoring; control of insertion depth.
	Nerve Injury	Very Low	Direct trauma or hematoma compression.	Immediate needle withdrawal; adjust puncture site/angle.
Early Postoperative	Pocket Hematoma	0.5% – 7.54%	Inadequate hemostasis; impaired coagulation.	Compression dressing; urgent explantation if expanding.
	Port Flip	0.4% – 0.5%	Large pocket; inadequate fixation; premature exertion.	Manual repositioning; surgical refixation if manual fails.
	Infection	1.5% – 4.7%	Breach in asepsis; cachexia; glucocorticoid use.	Strict asepsis; prophylactic antibiotics; may need explantation.
	Skin Breakdown	1.0% – 4.2%	Poor suture technique; mechanical friction; thin skin flaps.	Secondary suturing; explantation/debridement for contamination.
Late Postoperative	Catheter Occlusion	0.05% – 2.72%	Fibrin sheath; thrombus; drug precipitation; kinking.	Urokinase for thrombus; solvent agents for drugs.
	Catheter-Related Thrombosis	3.6% – 10.2%	Endothelial injury; hypercoagulability.	Standardized anticoagulation therapy (usually 3 months).
	CRBSI	2.7% – 8.2%	Poor maintenance; aseptic failure during access.	Antibiotic lock therapy; explantation for severe sepsis.
	Catheter Fracture	0.1% – 2.1%	Pinch-off syndrome; aging; increased abdominal pressure.	Diagnostic X-ray; prompt interventional/surgical retrieval.
	SVC Syndrome	0.2% – 1.7%	Catheter malposition; vigorous movement; thrombosis.	Diagnostic venography; repositioning or anticoagulation.
	Port-Site Metastasis	Rare	Tumor cell seeding; advanced malignancy.	Surgical resection, explantation, and adjuvant therapy.

CRBSI, Catheter-related bloodstream infection.

SVC, Superior vena cava.

TIVAP, Totally implantable venous access port.

Data compiled from clinical references (4, 5, 9, 10, 13, 28, 32, 47–62).

### 5.1 Intraoperative complications

Intraoperative complications typically arise from mechanical trauma during device implantation or acute physiological responses to the procedure.

#### 5.1.1 Pneumothorax

Pneumothorax is primarily caused by accidental pleural puncture or lung parenchyma injury by the needle tip, allowing air to infiltrate the pleural space. The reported incidence rate ranges from 0.2% to 1%, which is significantly influenced by factors such as the operator’s clinical experience, the choice of punctured vein, and the utilization of real-time ultrasound guidance. While routine post-operative chest radiography is standard, immediate imaging is mandatory if patients present with chest pain, thoracic tightness, or acute dyspnea. Management depends on severity: asymptomatic or stable, low-volume pneumothoraces may be managed conservatively with observation and supplemental oxygen. However, for severe or progressively worsening symptoms, thoracentesis or closed thoracic drainage is required to restore lung expansion (4, 5).

#### 5.1.2 Vascular injury

Accidental arterial puncture is a common complication of deep vein cannulation, occurring in approximately 1% of cases. While associated hemorrhage is usually self-limiting and manageable via manual compression, rare but severe consequences include large hematomas leading to airway compromise or pseudoaneurysm formation causing brachial plexus compression. To minimize these risks, clinical guidelines advocate for the combined use of ultrasound and fluoroscopic guidance to ensure precise anatomical orientation, the avoidance of multiple blind puncture attempts, and immediate post-procedural imaging to verify catheter positioning (47).

#### 5.1.3 Air embolism

Although extremely rare during venous port implantation, air embolism can be catastrophic. Air may enter the venous system through an open needle or sheath, especially if the patient is tachypneic or anxious. Clinical manifestations may include oxygen desaturation, sudden onset dyspnea, and a sense of impending doom; in severe cases, cardiovascular collapse or death may occur. The cornerstone of management is early detection and immediate cessation of further air entry. High-flow oxygen should be administered to facilitate air absorption and prevent pulmonary collapse. Furthermore, the patient should be placed in the left lateral decubitus position and Trendelenburg position (head-down) to trap air in the apex of the right ventricle, preventing its entry into the pulmonary artery (53).

#### 5.1.4 Arrhythmia

During venous port implantation, mechanical stimulation of the endocardium by the guidewire or catheter can trigger arrhythmias, most commonly atrial or ventricular premature contractions. Prolonged contact with the sinoatrial node or ventricular wall may precipitate life-threatening arrhythmia or even cardiac arrest. Consequently, continuous ECG monitoring is essential during catheterization, and strict attention must be paid to the insertion depth of the guidewire to avoid intracardiac irritation (54).

#### 5.1.5 Nerve injury

The incidence of iatrogenic nerve injury is remarkably low and is typically associated with direct trauma from repeated puncture attempts or secondary compression by a postoperative hematoma or the catheter itself. If a patient reports paresthesia or “nerve contact” sensations during the procedure, the needle must be withdrawn immediately. The puncture site or angle should be adjusted to avoid further blind attempts and potential permanent neurological deficit (5).

### 5.2 Early postoperative complications(≤30 days)

#### 5.2.1 Pocket hematoma

The incidence of pocket hematoma ranges from 0.5% to 7.54%. It primarily results from capillary hemorrhage during dissection of subcutaneous tissues, and is often associated with inadequate intraoperative hemostasis or impaired coagulation. The port can frequently be preserved if the hematoma is effectively managed; however, urgent explantation is required if the hematoma expands progressively or the risk of infection increases significantly (47–49).

#### 5.2.2 Port flip

Port flip is relatively rare when standardized implantation techniques are employed, with an incidence of approximately 0.4% to 0.5%. The main causes include an excessively large pocket, inadequate fixation, and premature upper limb exertion. Before implantation, the compatibility between skin elasticity, pocket size, and the port body should be carefully evaluated. Diagnosis can usually be confirmed by lateral chest radiography. Most cases can be successfully repositioned by manual manipulation; however, surgical reconstruction and refixation are necessary for cases refractory to manual reduction (4, 32).

#### 5.2.3 Infection

Infection is the leading cause of early port explantation. Risk factors include deviations from standard implantation procedures, cachexia, and long-term glucocorticoid use. The reported incidence ranges from 1.5% to 4.7%, significantly increasing unplanned explantation rates (47, 49). Preventive measures include preoperative evaluation of coagulation, meticulous intraoperative hemostasis, and local compressive dressing. For patients with established hematomas, ultrasonography should be performed promptly to rule out active bleeding. Additionally, strict adherence to aseptic techniques, regular postoperative dressing changes, and prophylactic antibiotics can reduce the risk of early postoperative infection.

#### 5.2.4 Local skin breakdown and wound dehiscence

Local skin breakdown and wound dehiscence are associated with suture techniques, mechanical friction, premature upper limb exertion, immunocompromise, malnutrition, and pharmacological factors. Thin skin flaps and compromised blood supply may also impair healing. The incidence is approximately 1.0% for arm ports and 4.2% for chest wall ports (55, 56). Early wound dehiscence caused by mechanical factors is characterized by discernible etiologies, typical symptoms, and mild local contamination, allowing for secondary suturing while preserving the port. In cases complicated by contamination, direct secondary suturing carries a high risk of recurrent infection; therefore, the port and catheter should initially be explanted, followed by debridement and suturing, with reimplantation considered after complete wound healing.

### 5.3 Late postoperative complications (>30 days)

#### 5.3.1 Catheter occlusion

The incidence of catheter occlusion ranges from 0.05% to 2.72%. It is characterized by loss of patency during infusion or injection, often manifesting as difficulty in blood aspiration. Common causes include catheter torsion or fracture, dislodgement or occlusion of the non-coring needle, fibrin sheath formation, and luminal obstruction due to thrombus formation or drug precipitation (48, 49, 51). When obstruction is encountered, malposition of the needle, as well as kinking in the non-coring needle or external tubing, should first be excluded. If catheter occlusion is highly suspected, chest radiography should be performed to verify the catheter course and tip position. In the absence of displacement, angiography or ultrasound may be used to evaluate for thrombosis or fibrin sheath formation. Mechanical obstruction requires inspection of the entire infusion system, adjustment of the catheter tip or patient position may be necessary prior to attempting recanalization. For drug-induced occlusion, the medication history should be carefully reviewed, and an appropriate thrombolytic or solvent agent selected according to the physicochemical properties of the involved drugs. Urokinase are the first-line treatment for thrombotic occlusion (32, 57).

#### 5.3.2 Catheter-related thrombosis

The incidence of catheter-related thrombosis ranges from 3.6% to 10.2%. Its occurrence is associated with multiple factors, including vascular endothelial injury, a hypercoagulable state, prolonged immobilization, and antineoplastic drug irritation. Asymptomatic thrombosis can be managed with observation; superficial venous thrombosis may be addressed symptomatically; however, symptomatic deep vein thrombosis requires standardized anticoagulant therapy in accordance with clinical guidelines. The anticoagulation course should typically be continued for 3 months following explantation (48, 49, 51).

#### 5.3.3 Catheter-related bloodstream infection

CRBSI is a major cause of premature TIVAP explantation or systemic sepsis, with an incidence of 2.7% to 8.2%. For patients with mild symptoms, port access should be temporarily suspended, and empirical antibiotics combined with high-concentration antibiotic lock therapy should be initiated alongside dynamic blood culture monitoring. For patients with severe symptoms (e.g., organ failure or isolation of specific pathogenic bacteria), the port system should be explanted as soon as possible, with systemic antibiotics selected according to antibiogram results. Prevention of CRBSI requires rigorous training in port maintenance, strict implementation of aseptic techniques, and regular febrile surveillance (48, 49, 52).

#### 5.3.4 Catheter fracture or dislocation

Catheter fracture is one of the most severe complications of TIVAP, primarily caused by improper catheter connection, aging, and pinch-off syndrome (accounting for approximately 40%). In addition, increased abdominal pressure due to forceful coughing or emesis can also induce dislodgement. Its overall incidence ranges from 0.1% to 2.1%. Chest radiography is the primary diagnostic method, though further angiography is required if damage is suspected. Once fracture or dislocation is confirmed, interventional or surgical retrieval should be performed promptly, unless the patient cannot tolerate the procedure (55, 58, 59).

#### 5.3.5 Catheter dislocation and superior vena cava syndrome

The incidence of catheter dislocation ranges from 0.2% to 1.7%, involving displacement into the subclavian vein, a branch of the superior vena cava (SVC), or upward into the internal jugular vein. This may impair infusion, blood aspiration, and management (58, 60). It is commonly caused by mechanical forces such as vigorous upper-body movement, fluctuating intrathoracic pressure, or port migration. Dislocation can induce thrombosis, fibrin sheath formation, and vascular stenosis. In severe cases, it may lead to SVC syndrome. SVC syndrome is a series of clinical manifestations resulting from venous flow obstruction. Incidence correlates closely with dislocation and thrombotic event rates (61). Conventional chest radiography remains the gold standard for diagnosis, providing visualization of tip position and any kinking. If inconclusive, computed tomographic angiography (CTA), echocardiography, or venography may be utilized. Venography is the gold standard for confirming SVC syndrome.

#### 5.3.6 Oncology-specific port-site metastasis

Port-site metastasis is a rare oncology-specific complication involving tumor cell seeding within the subcutaneous pocket or surrounding soft tissues. It is most prevalent in patients with advanced malignancies, hematologic, breast and lung cancers with high hematogenous metastatic potential. Treatment strategies should be individualized. For patients with favorable performance status (PS) and without extensive systemic metastasis, surgical resection combined with port explantation is recommended. Subsequent adjuvant therapy (chemotherapy, radiotherapy, or targeted therapy) should follow, guided by primary tumor histology. For patients with terminal disease treatment focuses on palliative care to improve quality of life (62).

### 5.4 Management standards and clinical practice

The cornerstone of mitigating TIVAP complications is predicated on a comprehensive, standardized full-lifecycle management framework. This approach encompasses strict adherence to aseptic protocols, accurate catheter tip positioning, routine postoperative surveillance, and prompt clinical intervention. Furthermore, thorough patient-centered education on device care, activity restrictions, and early symptom recognition is paramount. Multidisciplinary collaboration—integrating interventional radiologists, vascular access nurses, and clinical pharmacists—further maximizes device longevity and ensures reliable venous access throughout the continuum of care.

In the context of China, while several high-level expert consensuses have been established to guide practice, significant disparities persist in their standardized implementation across diverse healthcare tiers. Variability in institutional infrastructure, specialized training for vascular access teams, and the inconsistent adoption of real-time ultrasound guidance lead to notable inter-institutional differences in complication rates and procedural success.

To bridge this gap, our center (The Second Affiliated Hospital of Soochow University) has operationalized a standardized ultrasound-guided brachiocephalic vein (BCV) approach since 2017. Our technical refinements and clinical outcomes have been disseminated through several international publications, contributing to the global understanding of safe TIVAP implantation. Our data indicates that this approach achieves a high success rate (range: 92.52% -- 98.53%) and has effectively reduced perioperative complication rates by 71% compared to traditional landmark-guided methods. These internationally-published findings serve as a critical bridge between international standards (such as INS 2024) and advanced clinical practice in China, addressing the urgent need for standardized vascular access management nationwide.

## 6 Conclusion

TIVAP has emerged as a foundational supportive modality in contemporary oncology, revolutionizing long-term venous access by significantly enhancing treatment continuity and the overall patient experience. With its broadening clinical utility across diverse patient populations, adherence to evidence-based indications, selection of optimal implantation techniques and routes, and standardized full-lifecycle management are essential to minimizing adverse events.

Future research should prioritize the large-scale, multi-center prospective validation of promising access sites—particularly the ultrasound-guided brachiocephalic approach—to facilitate its broader clinical adoption. Additionally, the refinement of radiation-free positioning technologies (e.g., IC-ECG), and the development of bioactive, thromboresistant catheter materials. Such advancements will ultimately enhance the safety, efficacy, and global availability of TIVAP for all patients requiring prolonged venous access.

## Data Availability

The original contributions presented in the study are included in the article/supplementary material. Further inquiries can be directed to the corresponding author.
